# An Integrated Transcriptome-Wide Analysis of Cave and Surface Dwelling *Astyanax mexicanus*

**DOI:** 10.1371/journal.pone.0055659

**Published:** 2013-02-06

**Authors:** Joshua B. Gross, Allison Furterer, Brian M. Carlson, Bethany A. Stahl

**Affiliations:** Department of Biological Sciences, University of Cincinnati, Cincinnati, Ohio, United States of America; University of Maryland, United States of America

## Abstract

Numerous organisms around the globe have successfully adapted to subterranean environments. A powerful system in which to study cave adaptation is the freshwater characin fish, *Astyanax mexicanus*. Prior studies in this system have established a genetic basis for the evolution of numerous regressive traits, most notably vision and pigmentation reduction. However, identification of the precise genetic alterations that underlie these morphological changes has been delayed by limited genetic and genomic resources. To address this, we performed a transcriptome analysis of cave and surface dwelling *Astyanax* morphs using Roche/454 pyrosequencing technology. Through this approach, we obtained 576,197 Pachón cavefish-specific reads and 438,978 surface fish-specific reads. Using this dataset, we assembled transcriptomes of cave and surface fish separately, as well as an integrated transcriptome that combined 1,499,568 reads from both morphotypes. The integrated assembly was the most successful approach, yielding 22,596 high quality contiguous sequences comprising a total transcriptome length of 21,363,556 bp. Sequence identities were obtained through exhaustive blast searches, revealing an adult transcriptome represented by highly diverse Gene Ontology (GO) terms. Our dataset facilitated rapid identification of sequence polymorphisms between morphotypes. These data, along with positional information collected from the *Danio rerio* genome, revealed several syntenic regions between *Astyanax* and *Danio*. We demonstrated the utility of this positional information through a QTL analysis of albinism in a surface x Pachón cave F_2_ pedigree, using 65 polymorphic markers identified from our integrated assembly. We also adapted our dataset for an RNA-seq study, revealing many genes responsible for visual system maintenance in surface fish, whose expression was not detected in adult Pachón cavefish. Conversely, several metabolism-related genes expressed in cavefish were not detected in surface fish. This resource will enable powerful genetic and genomic analyses in the future that will better clarify the heritable genetic changes governing adaptation to the cave environment.

## Introduction

In nature, dramatic habitat shifts are frequently followed by rapid evolution of morphological and behavioral specializations [1]–[2]. In extreme environments, such as caves, these specialized phenotypes are striking, often including complete eye and pigmentation loss [3]–[9]. Interestingly, “cave-associated” phenotypes have evolved all over the globe, irrespective of phylogenetic position or local climate [10]–[13]. This convergence probably reflects, in part, the remarkably stable microenvironment of caves [4], which is conferred through elements such as a reduced nutrient base, narrow annual temperature and humidity ranges, and perpetual darkness [14]. These habitats also likely impose strong selective constraints that drive the convergence of cave-associated phenotypes [15].

Regressive phenotypes may evolve in cave-limited organisms because certain traits (vision, pigmentation) lose their utility in total darkness [16]. Thus, the gene(s) responsible for maintenance and expression of a phenotypic trait may tolerate the accumulation of loss-of-function mutations without fitness consequences for the organism [17]. Alternatively, maintaining useless traits in animals living amidst the depauperate cave environment may pose an energetic cost and therefore be selected against [14] [18]–[19]. A third possibility is that the pleiotropic consequence of adaptive genetic changes may indirectly interfere with the development of traits (e.g., eyes and pigment) that can be lost without fitness consequences for the animal [20]. Classical and contemporary studies provide mixed support for each of these hypotheses [17] [19] [21], however the precise genetic changes that lead to phenotypic losses in cave animals remain largely unknown [22].

A lack of available genetic resources has limited the identification of the precise genetic alterations that have accompanied the evolution of cave-adapted fish over the last several million years. Next-generation sequencing applications can provide powerful insights into the genetic constitution of new or emerging model systems [23]–[27]. In this study, we adapted Roche/454 pyrosequencing to the blind Mexican cavefish, *Astyanax mexicanus*. This animal is an excellent system for studying the genetic basis for cave-limited traits because conspecific cave and surface dwelling morphs can be found in the same region of northeastern Mexico [28]. Further, these morphotypes can be bred to produce viable hybrid individuals and large F_2_ pedigrees for linkage mapping and quantitative trait locus (QTL) analysis [29].

The aim of this project was to significantly expand the existing genetic tools for this emerging model system. This was carried out through deep pyrosequencing of normalized cDNA libraries derived from adult Pachón cave and surface dwelling individuals (Fig. S1). We assembled transcriptomes of cavefish sequencing reads alone, surface fish sequencing reads alone, and an integration of both sets of sequenced reads. Our integrated analysis produced the most successful assembly. This assembly enabled a direct method for evaluating conserved variation in 22,596 contiguous coding sequences (contigs; Fig. S2). Sequence variation discovered between cave and surface morphs was validated through a genotypic and linkage mapping analysis of 168 F_2_ individuals. Using the linkage map that resulted from this analysis, we confirmed the genetic basis of a Mendelian trait (albinism; [30]–[32]) using only 65 markers identified from our integrated transcriptome. Moreover, we successfully adapted an RNA-seq paradigm to identify “exclusive” genes that are expressed in cavefish but absent from surface fish, and vice versa. Using this approach, we observed that numerous vision-related genes (that are likely maintained at high levels of expression in surface fish) fail to be expressed in adult cave dwelling forms. These results may imply relaxed selection for genes involved in visual system maintenance in the cavefish lineage. Alternatively, the reduction (or suppression) of gene expression we observed may signal natural selection acting on the removal of vision in the cave-adapted organisms. Interestingly, we also discovered presence of a number of metabolism-related genes expressed in Pachón cavefish, whose expression was not detected in surface fish. This finding provides genetic support for the notion that the impoverished cave environment selects for metabolic efficiency in cave dwelling organisms.

Sequencing of the *Astyanax* transcriptome has dramatically expanded the available genomic resources for evaluating genetic changes in the blind Mexican cavefish. This dataset will facilitate future genetic and genomic studies investigating the dynamic nature of gene expression over the course of life history changes in *Astyanax*. Additionally, this project further demonstrates the significant amount of genomic synteny shared between *Danio rerio* and *Astyanax mexicanus* despite millions of years of divergence. Forthcoming studies will utilize and extend these resources to illuminate the genetic alterations that have accompanied adaptation to the subterranean environment over the past several million years.

## Results and Discussion

### An Integrated de Novo Transcriptome of Astyanax Mexicanus Derived from Cave and Surface Dwelling Fish Using Roche/454 Sequencing Technology

We produced three assemblies from our 454 sequencing analysis. The first assembly, using only Pachón cavefish-derived cDNA reads, yielded 15,292 contigs with an average coverage of 14 reads per contig. The second assembly, using surface fish-derived cDNA reads, yielded 13,022 contigs with a mean coverage depth of 13 reads. We reasoned that the evolved sequence differences between morphotypes were modest enough to allow a successful transcriptome assembly that incorporated both sequencing read pools. The resulting integrated analysis produced the most successful transcriptome assembly comprised of 22,596 contigs, with an average read coverage of 15. To minimize analysis of low-quality contigs, we applied a minimum depth cut-off of 10 reads as a criterion for subsequent contig construction and analysis. Our 10 reads-per-contig threshold provided the most manageable dataset for downstream analyses (i.e., manual BlastX queries, *Danio* positional identification).

**Figure 1:**
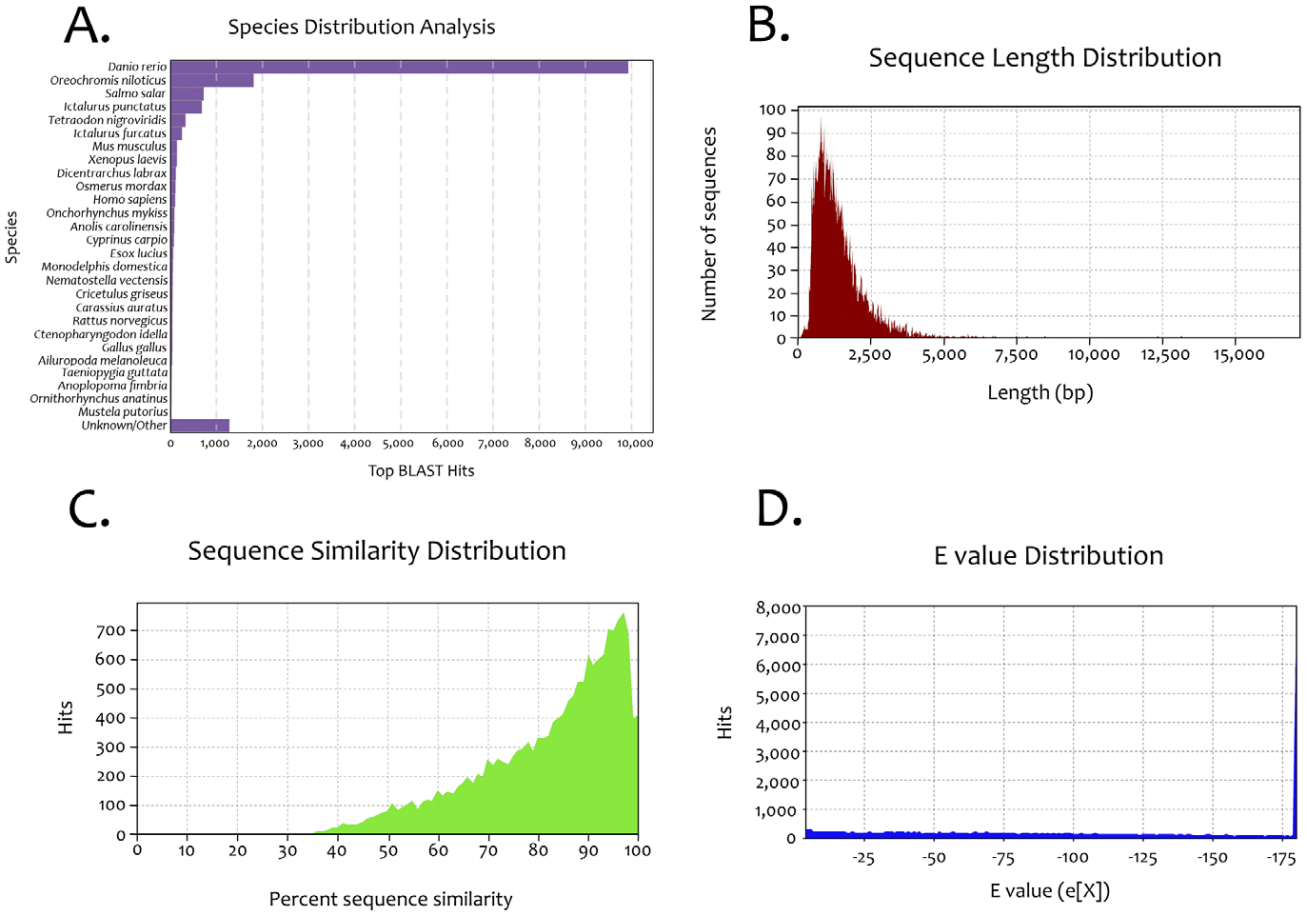
Sequence analyses of an integrated *Astyanax* transcriptome. The most common organism queried from our blast searches was the zebrafish, *Danio rerio* (A). The length of contigs produced from our *de novo* transcriptome assembly ranged from 47 bp to 16,952 bp (B), with an average size of ∼1,406 bp (Table 1). The returned sequence hits from our study showed a high sequence similarity based on the fact that the majority of returned hits harbored 90–100% sequence similarities to their respective “top” sequence hit (C). This high level of sequence similarity is also reflected in the distribution of “expect” values across all returned hits (D).

Our integrated *Astyanax* transcriptome, derived from both sets of sequencing reads, was analyzed using two methods. Our first approach involved manual queries of the consensus sequence for each contig using the NCBI-BlastX search tool (blast.ncbi.nlm.nih.gov). We performed a second analysis through an automated query of the same dataset using the online Blast2GO search tool (www.blast2go.com). This combined approach yielded high-quality and congruent datasets. For instance, both search tools indicated the most common organism queried was the zebrafish, *Danio rerio* (Fig. 1A). This result was not surprising since *Astyanax* and *Danio* are members of the Ostariophysian superorder [33], separated by ∼150 My. Despite this phylogenetic distance, we confirmed prior analyses [34] that indicated substantial conserved synteny between these two fish species.

The length of contigs produced from our *de novo* transcriptome assembly ranged from 47 bp to 16,952 bp (Fig. 1B), with an average size of ∼1,406 bp (Table 1). Collectively, the returned sequence hits of our integrated *Astyanax* transcriptome demonstrated a high sequence similarity based on the fact that the majority of returned hits harbored between 90–100% sequence similarity to their respective “top” sequence hit (Fig. 1C). Moreover, this high level of sequence similarity is reflected in the distribution of “expect” (E) values across all returned hits (Fig. 1D). Thus, the identity for each integrated contig in *Astyanax* could be assigned from each contig sequence despite having been created from two different morphotype pools.

**Table 1:** Comparison of transcriptome assemblies of Pachón cavefish, surface fish and integrated 454 sequencing reads.

		Pachón cavefish	Surface fish	Integrated
*Assembly Totals*				
	Contigs:	15,292	13,022	22,596
	Contigs >2 K:	2,230	1,654	4,037
	Contigs To Reach Calculated Genome Length:	6,282	5,669	11,832
	Assembled Sequences:	576,197	438,978	1,135,863
	Unassembled Sequences:	228,410	255,983	363,705
	All Sequences:	804,607	694,961	1,499,568
	Contig N50:	1,572 bp	1,521 bp	1,781 bp
	Average Coverage:	14	13	15
*Average Totals*				
	Sequences per Contig:	37	33	50
*Average Lengths*				
	Contigs:	1,331	1,295	1,406
	Assembled Sequences:	322	317	319
	Unassembled Sequences:	285	285	275
	All Sequences:	311	305	308
*Assembly Parameters*				
	Match Size:	21	21	21
	Match Spacing:	75	75	75
	Minimum Match Percentage:	85	85	85
	Match Score:	10	10	10
	Mismatch Penalty:	20	20	20
	Gap Penalty:	30	30	30
	Max Gap:	15	15	15
	Genome Length:	12,536,985	10,626,571	23,163,556
	Expected Coverage:	21	21	21
*Assembly Time*		0∶45∶34	0∶47∶23	1∶56∶26

**Figure 2:**
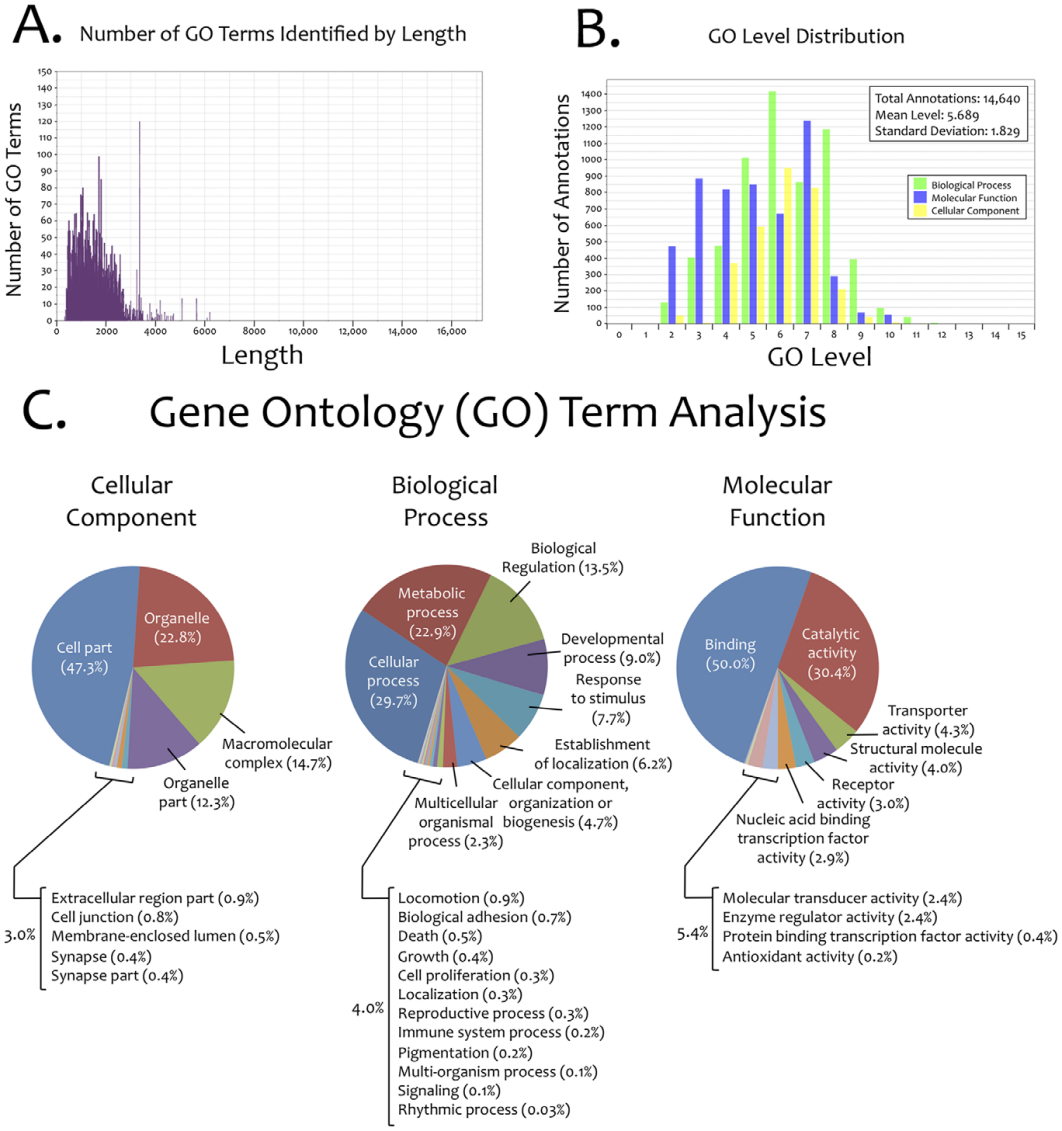
Gene Ontology (GO) term identification, annotation and distribution for an integrated *Astyanax* transcriptome. The majority of identified GO terms were assigned to contigs ranging from ∼200 bp–4000 bp in length (A). Each contig was assigned multiple GO term annotations encompassing the three principal GO categories (biological process, molecular function, cellular component). The overall distribution was similar for all three categories, with the largest number of GO term annotations assigned to 4–8 GO levels (B). We analyzed a subset of genes (n = 3,278) recognized from the *Danio* genome, and identified multiple sub-categories of “cellular components”, “biological processes” and “molecular functions” (C). The majority of “cellular component” terms represent “cell part” and “organelle”. The majority of “biological process” terms represent “cellular process”, “metabolic process” and “biological regulation”. The majority of “molecular function” terms represent “binding” and “catalytic activity”.

We next sought to determine the extent to which our integrated transcriptome could be assigned Gene Ontology (GO) terms based on assigned sequence identities. This analysis was carried out using the Blast2GO algorithm to successfully assign GO terms to the majority of contigs populating our integrated transcriptome (Fig. 2). The majority of GO terms were assigned to those contigs ranging in size from ∼200 bp–4000 bp in length (Fig. 2A). Within this dataset, each contig was assigned multiple GO term annotations in Blast2GO that encompassed the three principal GO categories (biological process, molecular function, and cellular component; Fig. 2B). The overall distribution of GO terms within our dataset was analyzed using a subset of 3,278 contigs that demonstrated the highest level of sequence similarity to genes to *Danio rerio* (Fig. 2C). This subset analysis suggests our integrated transcriptome reflects a broad distribution of genes (reflected by the diverse categorical representation of GO terms) expressed in adult *Astyanax mexicanus* surface and cave morphs (Fig. 2C).

### Genetic Variation between Surface and Cave Dwelling Individuals was Readily Identified through our Integrated Transcriptome

**Figure 3:**
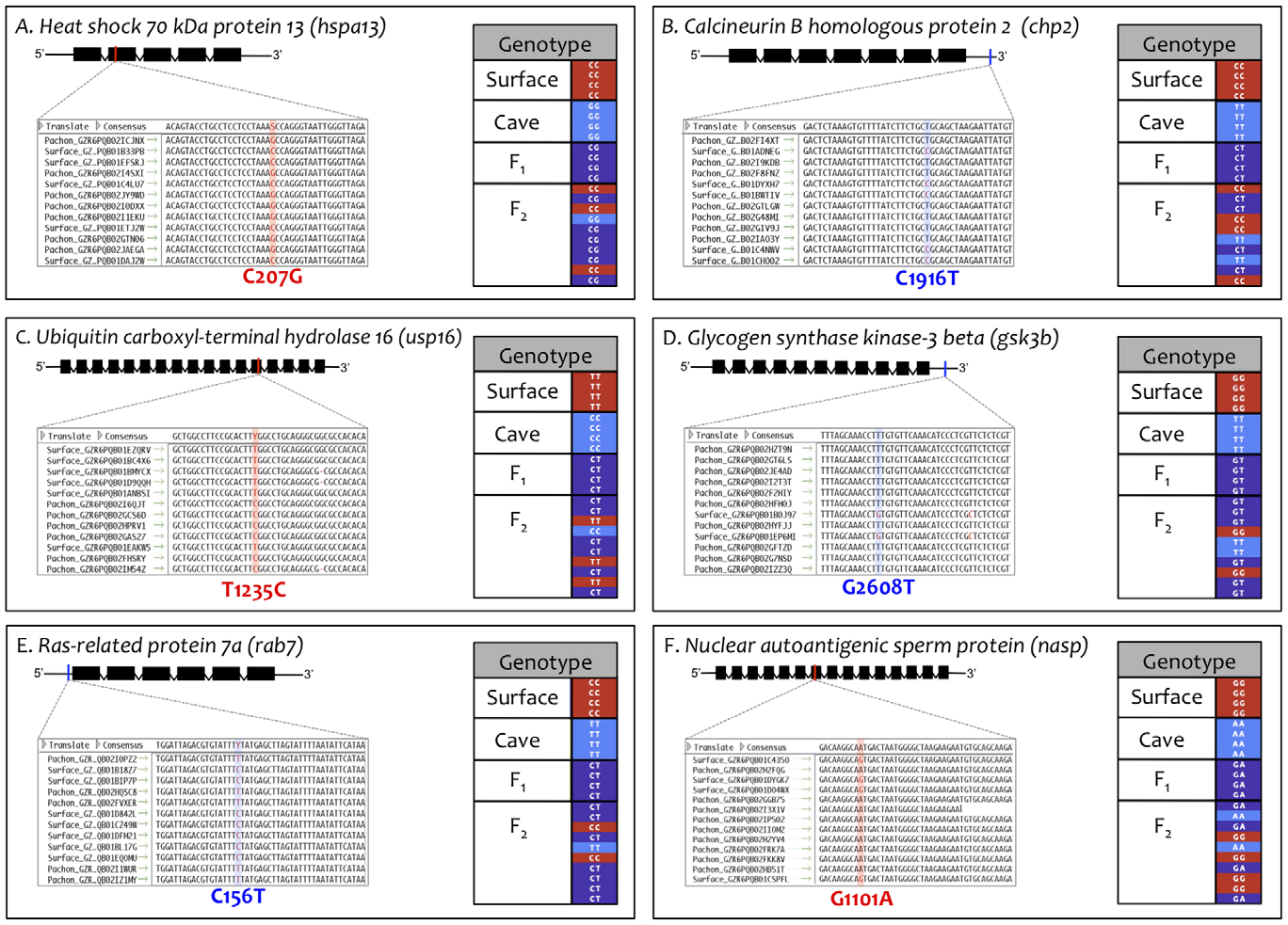
SNP identification yields numerous markers for genotypic studies. Several genes with relevant positional information spanning 25 *Danio rerio* chromosomes were mined for SNPs segregating between *Astyanax* surface and cave morphotypes. SNP locations were categorized as either coding (red) or non-coding (blue). SNP markers in six genes were assembled into three *Astyanax* linkage groups that anchored to four chromosomes in *Danio rerio*: LG5/Chr15 (A–C), LG13/Chr9 (D), LG14/Chr11 (E), and LG14/Chr6 (F).

Genetic variation between cave and surface dwelling individuals was clearly observed within our dataset. However, in some cases, we could not rule out sequence variation resulting from the inclusion of low quality sequence reads into a particular contig. To minimize analysis of false positives, we assigned a minimum depth coverage cut-off value of 10 reads or higher to each contig (Materials and Methods). This filter ensured that only those sequence polymorphisms with reasonably strong support advanced to our genotypic analyses. To further assess the validity of potentially informative sequence variations, we analyzed a subset of contigs demonstrating sequence variation in a set of highly conserved genes (Fig. 3). Each gene was selected based on the genomic position of the orthologous gene in *Danio rerio*. In each case, the identity and position of a particular SNP was categorized as either located within the coding sequence (Fig. 3A, 3C, 3F) or the non-coding region of a given gene (Fig. 3B, 3D, 3E).

The genomic position of orthologous genes in *Danio* was determined using the MapViewer function in the Entrez-Gene database at the National Center for Biotechnology Information (Methods). Using this method, a total of 7,806 contigs (∼34.5%) were identified from our integrated *Astyanax* transcriptome that mapped to an orthologous position in *Danio rerio*. The distribution of the number of orthologous genes identified within our integrated transcriptome (y-axis; Fig. S3A) by chromosomal number in *Danio rerio* (x-axis; Fig. S3A) was uneven. We sought to determine if this distribution reflected a bias in our transcriptome assembly or simply the number of genes on each chromosome in *Danio*. We assessed the correlation between the number of positive returned hits for a given chromosome in *Danio* (x-axis; Fig. S3B) and the number of identified genes on each chromosome in *Danio* (y-axis; Fig. S3B). Based on this analysis, these two variables demonstrated a significant positive correlation (r = 0.578, p = 0.002; Fig. S3B) suggesting that more genomic positions were identified on *Danio rerio* chromosomes that are more heavily populated with genes. We believe this result reflects that our transcriptome represents a fairly even distribution of genes present in our integrated assembly.

### Genetic Linkage Analysis Reveals Synteny between the Danio Genome and a Transcriptome-based Astyanax Linkage Map

Albinism is a Mendelian trait in Pachón cavefish [30]. Protas et al. (2006) identified *Oca2* as the causative albinism locus based on a QTL study of 254 markers in an F_2_ pedigree of Pachón cave x surface fish [26]. A *post-hoc* study of the genomic sequences flanking 8 microsatellite markers used by Protas et al. (2006) localized to chromosome 6, upon which *Oca2* resides in *Danio* [34]. This study observed a number of syntenic stretches that united significant distances in *Asytanax* (in cM) to significant distances in *Danio* (in Mb). Herein, we identified genes based on their positions in *Danio* to determine if these genes reside on the same linkage group(s) in *Astyanax*.

**Figure 4:**
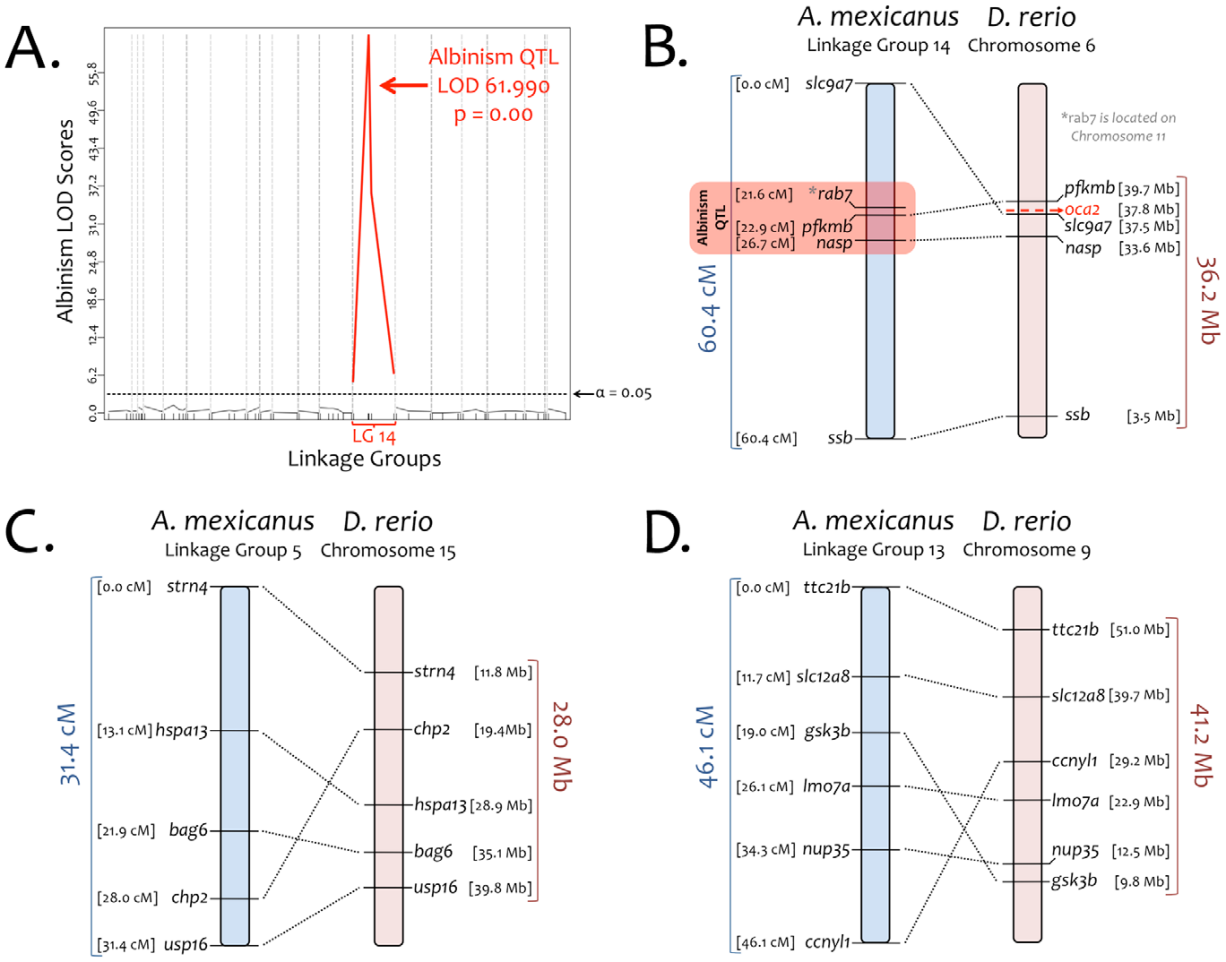
Genotypic and linkage analysis confirms regions of synteny between *Astyanax* and *Danio*. We anchored a low-density *Astyanax* linkage map (based on 65 markers in 168 individuals) to the *Danio* genome. These syntenic stretches included *Astyanax* linkage group 5 spanning ∼31.4 cM which corresponds to a ∼28.0 Mb stretch of *Danio* Chromosome 15 (11.8–39.8 Mb) wherein the homologous genes reside (A). Similarly, linkage group 13, spanning 46.1 cM in *Astyanax*, included six markers corresponding to a ∼41.2 Mb portion of *Danio* Chromosome 9 (9.8–51.0 Mb; B). Linkage group 14 consisted of five markers encompassing ∼60.4 cM in *Astyanax* (C). Four of five genetic markers were located within a ∼36.2 Mb region of *Danio* chromosome 6 (3.5–39.7 Mb). Within this genomic block in *Danio* was *Oca2*, which causes albinism in *Astyanax* [29]. A QTL analysis of albinism in our F_2_ mapping pedigree (D) identified a highly significant QTL peak (LOD score = 61.990) on linkage group 14, providing evidence of synteny between this linkage group and *Danio* chromosome 6.

Our analysis, sampling only 65 markers in 168 individuals, identified numerous syntenic blocks between 17 chromosomes in *Danio* and 20 linkage groups in *Astyanax* (Fig. 4). We attribute unrepresented chromosomes in *Danio* to an incomplete genotypic analysis as well as our small number of F_2_ individuals. Despite this, we were able to successfully map the (binary) albinism phenotype within our pedigree. Our analysis revealed a single, highly significant QTL (LOD value = 61.9), as similarly demonstrated by prior studies [29], that resides on linkage group 14 (Fig. 4A). Of the five markers present on this linkage group, four (*slc9a7*, *pfkmb*, *nasp*, *ssb*) are present on chromosome 6 in *Danio rerio* (Fig. 4B). One gene, *rab7*, is localized to chromosome 11 which may reflect the presence of a paralogous gene that resides at this genomic position in *Astyanax*, the translocation of this gene from an ancestral position to its current position in *Astyanax* relative to *Danio*, or inaccurate genotypic analysis.

In sum, we detected the presence of four genes that encompass a ∼36.2 Mb genomic interval and co-localize to chromosome 6 (along with *Oca2*) in *Danio* (Fig. 4B). Additional stretches of synteny were similarly identified from 20 linkage groups that were successfully anchored to 17 chromosomes (Fig. 4C, 4D). These observations offer further support for the presence of numerous conserved (syntenic) blocks between the genomes of *Danio* and *Asytanax*. A recent study demonstrated efficient mapping of mutations in zebrafish using whole-genome sequencing approaches [35]. Our results suggest that the combination of a large panel of informative SNPs (such as those identified through our transcriptome study) along with whole genome sequencing technology will enable similar approaches to be adapted in *Astyanax*.

### Adaptation of Astyanax Roche/454 Sequencing Reads to an RNA-seq Paradigm

**Figure 5:**
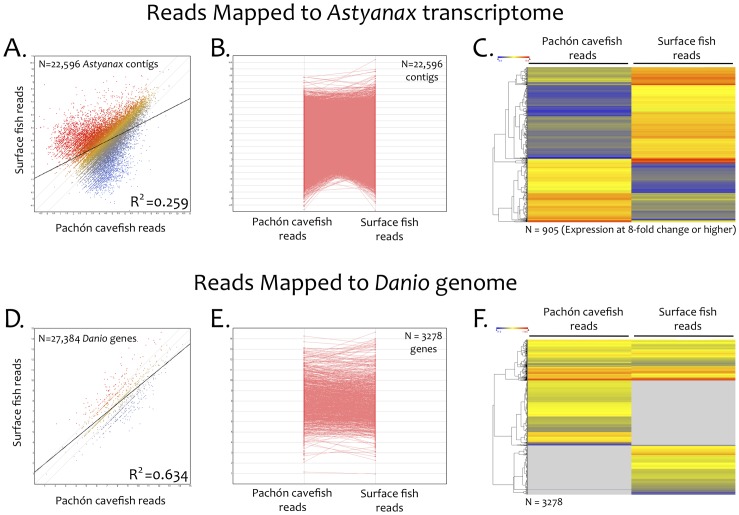
Adaptation of an RNA-seq approach for coordinate expression analyses in *Astyanax mexicanus*. We adapted an RNA-seq strategy to analyze transcripts represented highly in the surface cDNA pool compared to cavefish (red, A), transcripts represented sparsely in surface compared to cavefish (blue, A), or transcripts expressed at equivalent levels in both morphotypes (yellow, A) using our integrated *Astyanax* transcriptome as a template (A–C). This analysis revealed a reduced level of correlated gene expression between morphotypes (R^2^ = 0.259), indicating measurable differences in expression despite our use of normalized cDNA libraries. A line graph representation of expression level similarly demonstrates mRNA expression level differences between morphotypes (B). A heat-map analysis of genes expressed at an 8-fold difference between morphotypes identified a large number of genes (n = 905) with contrasting expression patterns (C). Several genes identified from our integrated transcriptome analysis were adapted for use as markers in a linkage map (see Fig. 4). The same analysis was implemented using the *Danio rerio* genome as a template (D–E). This study enabled successful identification and expression analysis of 3,278 genes, using the same parameters as those utilized for the integrated transcriptome as a template (D). Although the number of genes analyzed was far fewer with this approach, correlated expression patterns of these genes were higher between morphotypes (R^2^ = 0.634). Line graph and heat map representations of these genes (E, F) demonstrate *Astyanax* transcript reads can be successfully mapped to the *Danio* genome.

We evaluated our dataset using an RNA-seq strategy in which we mapped the sequencing reads derived from the cave- and surface-specific libraries to each of two templates. Our first analysis involved mapping cave and surface reads to our integrated transcriptome template (Fig. 5A–C). This template consists of a fasta-formatted file denoting each of the complete contig sequences along with the name of the gene identified through our BlastX queries. While this approach is not informative with respect to comparative gene expression level (because each morphotype-specific library was created from normalized libraries), it did permit discovery of genes expressed exclusively within each morphotype. We defined “exclusive genes” as those expressed (for example) in the cavefish cDNA pool, but absent from the surface fish cDNA pool. We found that every read from our Roche/454 analysis mapped to the integrated transcriptome.

The distribution of reads from this study demonstrated significant numbers of genes with high read counts in surface fish/low read count in cavefish (Fig. 5A, red), genes with high read counts in cavefish/low read count in surface fish (Fig. 5A, blue), and sets of genes with roughly equivalent read counts in both morphotypes (Fig. 5A, yellow). We further analyzed this dataset by measuring the linear expression plot by each morphotype. Despite having used normalized libraries, we could plot the relative expression of genes expressed at an 8-fold level or higher (n = 905) in one morphotype compared to the other morphotype (Fig. 5C). This could be attributed to the fact that significant differences in gene expression are present between morphotypes for this gene set. Forthcoming analyses are essential for determining the relevance of this preliminary result. However, our analysis provides evidence that non-normalized libraries derived from morphotype-specific cDNA pools can be compared using a robust, transcriptome-wide approach for expression level comparison.

Next, we performed a parallel analysis by mapping *Astyanax* sequencing reads to the published *Danio rerio* genome template (Fig. 5D–F). Far fewer *Astyanax* sequencing reads mapped to the *Danio* genomic template compared to our integrated transcriptome (3,278 compared to 22,596). We attributed this reduced success to two factors. First, our cDNA sequencing reads were derived from RNA pools, and therefore many *Astyanax* reads were anticipated to fail to map to *Danio* genomic regions that abut or cross intronic boundaries. Second, failure of *Astyanax* reads to map to *Danio* may be explained by evolved sequence differences between the two teleost fish lineages. In light of this, the fact that >3,000 genes did successfully align from our sequencing project indicates a sizable amount of sequence conservation between *Danio rerio* and *Astyanax mexicanus*. This sequence conservation further supports the notion of significant genomic synteny shared between the two Ostariophysian lineages [34].

### Numerous Genes Expressed in One Morphotype Fail to be Expressed in the other Morphotype

**Figure 6:**
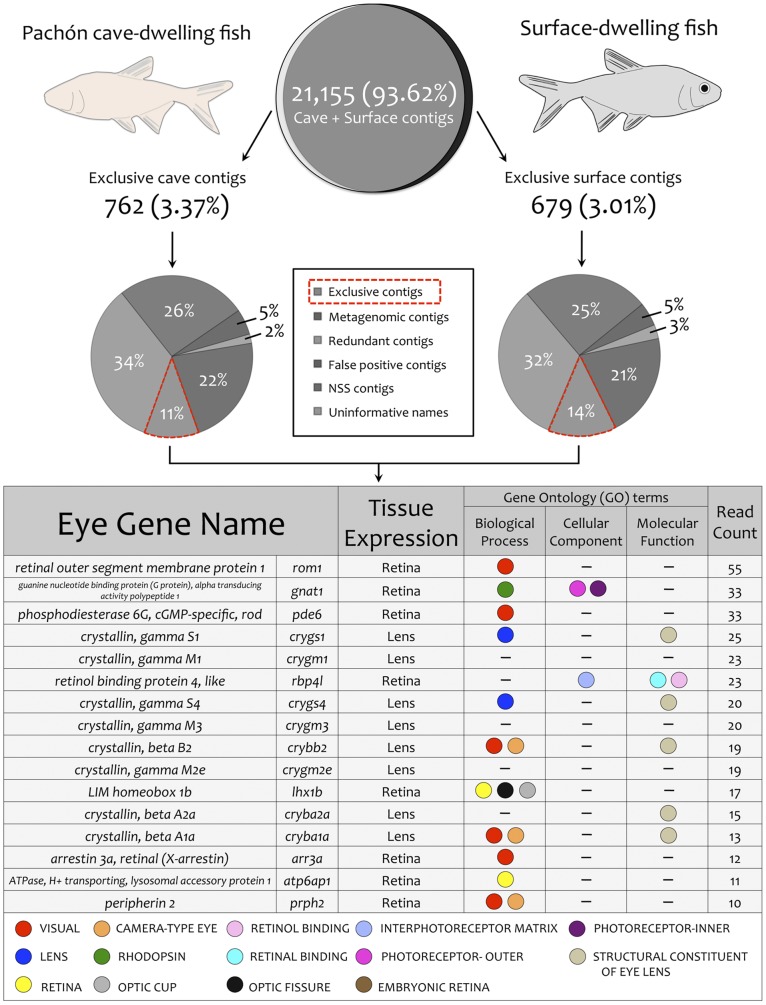
An analysis of exclusive contigs reveals a number of visual system genes not expressed in adult cavefish. The vast majority (∼94%) of contigs in our integrated transcriptome were assembled from cave and surface cDNA sequences. The remaining ∼6% were exclusively derived from either the cavefish cDNA pool (n = 762) or the surface fish cDNA pool (n = 679). We filtered this dataset and evaluated contigs that were informative and expressed in an exclusive fashion (red dashed line). This approach revealed numerous eye genes are exclusively expressed in surface fish, including a number of *crystallin* genes essential for lens production and maintenance.

A secondary analysis of our integrated transcriptome revealed that ∼94% of the 22,596 contigs were assembled from reads comprised of both cave and surface morphotype-specific sequences (Fig. 6). We reasoned that the identification of exclusively-expressed contigs may reveal genes of ecological relevance. We identified a set of genes derived exclusively from the cavefish cDNA pool (n = 762) as well as the surface fish cDNA pool (n = 679) and noted that many could be sub-categorized (Fig. 6). For instance, 26% and 25% of the contigs derived from the exclusive cavefish versus surface fish read pool, respectively, demonstrated no significant similarity (NSS) to sequences accessioned to NCBI (Fig. 6). We further filtered (see Materials and Methods) each exclusive set to determine those genes of *known* identity that were represented in one morphotype cDNA pool but not in the other. This resulted in a subset of ∼11% of the 762 Pachón cave contigs and ∼14% of the 679 surface contigs that were subsequently analyzed based on function, as determined from a GO term analysis.

**Figure 7:**
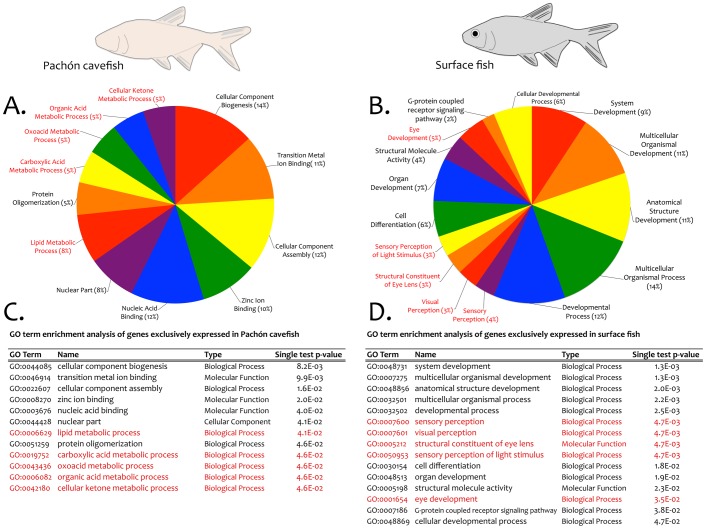
GO term enrichment analyses of exclusive contigs derived from adult cavefish and surface fish. The exclusive contig set derived from Pachón cavefish (A, C) is significantly enriched for a number of genes, including those associated with metabolism (yellow highlight in A, C). An analysis of exclusive surface fish contigs revealed a wider diversity of enriched GO terms (B, D), including several categories associated with vision (yellow highlight in B, D).

We performed an analysis for GO term enrichment in each exclusive contig set. We identified a number of GO terms in adult Pachón cavefish that are enriched for metabolism-related processes (red; Fig. 7A, 7C). This finding is supported by a number of historical reports indicating cave-adapted organisms frequently harbor a more efficient metabolism [36]–[39]. These changes likely evolve as an adaptive response to impoverished nutrient supply within the cave microenvironment [40]. Specifically, prior studies established that *Astyanax* cavefish demonstrate reduced oxygen consumption compared to surface dwelling morphs [41]. Based on ultrastructural analyses of the pineal gland, Omura (1975) predicted changes in protein and lipid metabolism in cave dwelling *Astyanax* morphs [42]. Consistent with this prediction, we noted enrichment for the GO terms: “lipid metabolic process” as well as “protein oligomerization” (Fig. 7A, 7C). In addition, our finding of enrichment for the GO term “cellular ketone metabolic process” supports a recent study by Salin et al. (2010) that identified a higher activation of ‘compensatory’ metabolic pathways (including ketogenesis) in *Astyanax* cavefish [43]. In sum, our analysis provides genetic support for the notion of adaptive genetic changes that have evolved in cavefish to enable a more efficient metabolism in the subterranean environment.

Our enrichment analysis also indicated a more diverse set of GO terms in surface fish (15 GO terms compared to 12 GO terms in cavefish). Interestingly, this analysis revealed enrichment for seven GO terms associated with development (Fig. 7B, 7D). In addition, five GO terms from this enrichment analysis were associated with visual system development, function and structure (red; Fig. 7B, 7D).

### Multiple Visual System Genes are not Expressed in Adult Pachón Cavefish

The reduction of visual system gene expression identified from our analysis was not surprising since Pachón cavefish begin to develop an eye during embryogenesis that regresses as they advance to juvenilehood [5]. Our study, however, bolsters support for the role of certain genes in eye regression in *Astyanax* cave forms. For instance, one of the genes we identified that is most strongly expressed in surface fish, and absent from cavefish, is *rod outer membrane segment 1* (*rom1*). This gene was previously found to reside near an eye-loss QTL identified from a surface x Pachón F_2_ pedigree analysis [34]. The *rom1* gene product confers structural integrity to the outer discs of rod cells [44]. In the absence of *rom1* (and *prph2,* also identified from our analysis), rods undergo degeneration and lead to retinal degeneration in RDS mutant mice [45]–[46]. The combination of genetic and expression data provided here further suggests a role for this gene in the heritable loss of the eye in Pachón cavefish.

Another gene demonstrating high levels of expression in surface fish, but absent from cave forms, is *gnat1*. This gene encodes a G-protein responsible for coupling rhodopsin and cGMP-phosphodiesterase during visual system impulse propagation in rod cells [47], [48]. Strickler and Jeffery (2009) similarly demonstrated a strong reduction of *gnat1* expression in cavefish compared to surface fish using a cross-species *Danio* microarray approach [49]. In contrast to our study, Strickler and Jeffery (2009) analyzed RNA pools isolated from 3-day old embryos [49]. Therefore, collectively our findings suggest that certain genes exhibiting reduced expression during embryogenesis may no longer be expressed as cavefish advance to adulthood.

We identified other retinal genes demonstrating significant differences in expression, including the rod-specific phosphodiesterase, *pde6*, the retinol binding protein, *rbp4l*, the lim-domain homeobox, *lhx1b*, and the ATPase, *atp6ap1.* While none of these genes were specifically identified using their cross-species microarray analysis, Strickler and Jeffery (2009) observed significant down-regulation of a phosphodiesterase (*pde4b*) and ATPase (*ATPase V1-D*), both of which are closely related to the absent genes identified in our study [49].

In *Astyanax,* the lens plays a critical role in promoting cell survival in the developing eye [50], and influences development of the optic nerve and tectum [51]. We noted absent expression of a number of lens-specific markers, including eight *crystallin* genes (*crygs1*, *crygm1*, *crygs4*, *crygm3*, *crybb2*, *crygm2e*, *cryba1a*, and *cryba2a*). These observations are congruent with the early work of Langecker et al. (1995) who discovered γ*_s_-crystallin* was not transcribed during late stages of development in cave forms [52]. Behrens et al. (1998) subsequently characterized the genetic structure of a different *crystallin* gene, α*A-crystallin*, and found that despite an intact coding sequence in cave dwelling fish, the expression of this gene was never detectable using *in situ* hybridization [53]. Thus, the lack of *crystallin* gene expression in adult Pachón cavefish is likely the case for most (if not all) *crystallin* family members, and reflects the central role that high levels of expression of these proteins play in lens maintenance and survival [54]–[57].

In light of transcriptome analyses carried out in other cave-limited species, it was notable that we did not detect expression of the gene *arrestin 3a* (*arr3a*) in adult Pachón cavefish. This gene product is responsible for quenching photoactivated rhodopsin [58] in mature rod cells. The expression of two close *arrestin* family members (*arrestin 1* and *2*) was recently observed in a transcriptomic analysis of the Mammoth Cave beetle, *Ptomaphagus hirtus*. The presence of *arrestin* gene expression, along with a measurable phototactic response, indicated preservation of phototransduction in this cave-limited species [26]. However, here we report undetectable expression of an *arrestin* gene in cave dwelling fish. This result may indicate different genes, in vastly different taxa, are subject to differential reduction or suppression of expression in the cave environment.

### Conclusions

In this report, we present the results of a deep pyrosequencing analysis of Pachón cavefish and surface dwelling morphs of *Astyanax mexicanus*. Using this strategy, we successfully assembled the first high-quality *Astyanax* transcriptome using next-generation sequencing technology. This integrated assembly enabled discovery of informative sequence variation for thousands of contiguous coding sequences. We validated this sequence variation through a genetic linkage analysis of albinism carried out in surface x cave F_2_ individuals. We further mined our dataset using an RNA-seq paradigm to identify “exclusive” genes expressed in either cavefish or surface fish. We discovered numerous vision-related genes expressed strongly in adult surface fish, which are not detected in adult cavefish. Conversely, we identified numerous metabolism-related genes expressed strongly in adult Pachón cavefish, which are not detected in adult surface fish. Additionally, we further characterized significant genomic synteny between *Danio rerio* and *Astyanax mexicanus* despite ∼150 million of years of divergence.

This study significantly expands the available genetic tools for *Astyanax mexicanus* and will enable future genetic and genomic studies investigating the dynamic nature of gene expression over the course of life history. These investigations will lend powerful insight to gene expression differences that accompany developmental changes, spatial/localization differences, or responses to environmental perturbations in *Astyanax*. These studies will, in turn, help inform our understanding of the relative roles of selective versus neutral forces in the evolution of regressive phenotypes in cavefish. By utilizing and extending the resources presented here, future analyses will help determine the global genetic alterations that accompany adaptation to the subterranean environment.

## Materials and Methods

### Ethics Statement

This study was carried out in strict accordance with the recommendations in the Guide for the Care and Use of Laboratory Animals of the National Institutes of Health. The protocol was approved by the Institutional Animal Care and Use Committee (IACUC) of the University of Cincinnati (Protocol Number 10-01-21-01). All animals were anesthetized using tricaine methanesulphonate (MS-222), and every effort was made to minimize suffering.

### cDNA Library Preparation and Sequencing

We generated total RNA from four individuals: one male and one female adult cavefish derived from the Pachón cave population, and one male and one female adult surface dwelling fish (*Astyanax mexicanus*). All surface and Pachón cavefish were reared under identical conditions. Both female and male individuals were members of the same pedigree, and reared in groups of five individuals within 5-gallon tanks. Our lab husbandry unit (Aquaneering, San Diego, CA) is equipped with multiple filters (i.e., carbon, UV, micron and dense particulate filters) and each tank receives fresh, recirculated conditioned water (pH: ∼7.4; conductivity: ∼800 µS). All fish were maintained under a 12 hr light:12 hr dark photic schedule and fed tetra flake food (TetraMin Pro) daily. High-quality total RNA was isolated from each whole fish using the *mir*Vana miRNA Isolation Kit (Ambion; Grand Island, NY) according to the manufacturer’s instructions. The male Pachón cavefish prep (∼22.4 µg/150 µl) and the female Pachón cavefish prep (∼36.7 µg/150 µl) were combined into one Pachón cavefish-specific pool (∼59.1 µg/300 µl). Similarly, the male surface dwelling fish prep (∼24.0 µg/150 µl) and the female surface dwelling fish prep (∼26.4 µg/150 µl) were combined into one surface fish-specific pool (∼50.4 µg/300 µl). Each pool was sent to Eurofins MWG Operon (Huntsville, AL) on dry ice for library preparation and sequencing.

The total RNA samples were quality analyzed with a Shimadzu MultiNA microchip electrophoresis system (Fig. S1A). The normalized libraries were generated from 41.4 µg of the surface fish pool (S28/S18 ratio = 1.6) and 52.1 µg of the Pachón cavefish pool (S28/S18 ratio = 1.5). First-strand cDNA synthesis was carried out on poly(A)+ RNA isolated from the total RNA samples (Fig. S1B). Pools of RNA were primed using an N6 randomized primer. 454 adapter sequences were then ligated to the 5′ and 3′ ends of the cDNA. A short barcode sequence (Pachón cave = TCTACT; surface = TCGTAT) was added to the 5′-adapter sequence to identify the origin of each read for subsequent sequencing assembly and analysis. The cDNA was then amplified by PCR (14 cycles) using a proof-reading enzyme. cDNA was normalized (Fig. S1B) using one cycle of denaturation and reassociation. Following reassociation, double-stranded cDNA was separated from single-stranded cDNA using hydroxyapatite column separation. Following separation, single-stranded cDNA was PCR amplified for 7 cycles. cDNA pools in the range of 500–800 bp were extracted from an agarose gel and prepared for sequencing (Fig. S1C). Rapid high throughput sequencing was performed in a full run using Titanium series chemistry on the Roche GS FLX system (Eurofins MWG Operon; Huntsville, AL).

### de Novo Transcriptome Assembly

All reads from the cave fish-specific and surface fish-specific pools were provided in fasta format in both raw and clipped versions. Clipped versions were used for subsequent *de novo* transcriptome assembly. These versions of our reads were modified for the removal of the barcode sequences used to identify each morphotype (see Methods) and were processed for a high-accuracy quality score. To minimize analysis of miscall errors [59] during our transcriptome assembly, we applied a quality score cut-off of 20 (Q20), which provided an accuracy base-call value of 99% [60]. Every read collected from the sequencing run (1,499,568 reads) was included in our assembly, however 363,705 reads did not assemble using the NGen software package and were categorized as singletons. Every read in each fasta file was tagged with the prefix “Pachon-“ or “Surface-“ according to the origin of the reads. Three assemblies were produced: a Pachón read assembly, a surface read assembly, and an integrated assembly that integrated both the Pachón and surface reads. Each assembly was created using SeqMan NGen (v.3.0.4; DNAStar, Madison, WI).

We utilized SeqMan NGen software for our *de novo* transcriptome assembly because of its strength in recapitulating known transcripts, producing of the highest number of novel sequences, and demonstrating the highest overall contig assembly performance compared to other assemblers using Roche/454 and Illumina datasets [61], [62]. Assemblies were created *de novo* according to the following default parameters: match size = 21; match spacing = 75; minimum match percentage = 85; match score = 10; mismatch penalty = 20; gap penalty = 30; max gap = 15. The results of each assembly are presented in Table 1.

We tested the robustness of our integrated assemblies using three different ‘minimum match percentage’ values. The optimal value for Roche/454 reads, according to SeqMan NGen, is 85%. We also tested 75% and 95% minimum match percentages. A minimum match percentage of 75% produces ∼500 fewer contigs overall, and ∼40 fewer contigs that were greater than 2,000 bp in length. This latter metric is valuable for estimating the number of ‘complete’ transcripts (i.e., inclusive of the open reading frame and much of the 5′ and 3′ untranslated regions) identified from our analyses. A minimum match percentage of 95% produced far fewer contigs greater than 2,000 bp in length compared to the 85% assembly. Moreover, the 95% minimum match parameter left far more (>200,000) sequences unassembled and produced an average contig length of only 1,145 bp compared to 1,406 bp for the 85% assembly (Table 2). The 85% match criterion we used produced the best overall assembly statistics, including the highest contig N50 value of 1,781 bp (compare to 1,771 bp for 75%; and 1286 bp for 95%).

**Table 2:** Descriptive analysis of the Astyanax integrated transcriptome assembly.

	Contig Length	Max Score	Total Score	Query Coverage	Maximum Identity
**Count**	22,596	19,438	19,438	19,438	19,438
**Sum**	31,769,005	5,946,226.8	9,083,122.01	N/A	N/A
**Mean**	1,405.96	305.91	467.29	53.35%	70%
**Median**	1,201	225	279	54%	75%
**Min**	47	5.1	12	1%	9.00%
**Max**	16,952	5,144	103,400	100%	100%
**Standard Deviation**	851.70	294.78	1,358.94	0.26	0.21

### Blast Analyses and Transcriptome Annotation

The probable identity of each contig from our integrated transcriptome assembly was queried manually using the National Center for Biotechnology Information (NCBI) database. We utilized the BlastX algorithm to assess the identity of the most closely related gene in other organisms. All sequence identities were catalogued to a spreadsheet with the following information recorded for each contig sequence: accession number, gene description, max score, total score, query coverage, e value, and maximum identity value (Table S1). In instances where the name of the gene was defined as “unknown” or “hypothetical”, the next recognizable name in the returned hit was recorded as an alternate name. For all genes with a top hit returned in the zebrafish (*Danio rerio*) the position of the gene (i.e., the MapViewer position) within the Zv9 draft of the zebrafish physical genome was recorded.

A parallel analysis of sequence identities was performed using the online tool Blast2GO (www.blast2go.org). These analyses were carried out using the NCBI blast server and non-redundant (nr) database. For each contig, the top blast hit was collected, assuming an expect value cut-off of 1.0×10^−3^. Searches were carried out using the BlastX program and the QBlast-NCBI mode with a low complexity filter and an HSP length cut-off of 33.

Blast2GO provided information for several transcriptome-wide parameters, including: blast searches (species distribution analyses of blast searches, contig sequence length distributions, sequence similarity distributions) and expect (E) value distributions; Gene Ontology (number of GO terms identified by length and GO level distributions); annotation results (percentage of sequences annotated by length, annotation score distributions and evidence code distributions; Fig. S4A–C). We performed an enrichment analysis of exclusively-expressed genes using Blast2GO. We first created a combined annotation file set comprised of all adult cavefish and surface fish exclusive contigs (n = 111) for which GO terms were identified. We then assessed all genes derived exclusively from Pachón cavefish (“test-set”) compared to all genes derived exclusively from surface fish (“reference”), and vice versa. We performed this analysis using a term filter value of 0.05 and the p-value term filter mode. This test indicated if GO terms were enriched in a test group compared to a reference group using Fisher’s Exact Test with multiple testing, controlling for the false discovery rate [63].

We filtered our integrated transcriptome to estimate the total number of known genes identified from this study. Of the 22,596 total contigs we assembled, 3,158 did not return a hit to a known sequence. Of the remaining 19,438, we removed all sequences that were either redundant or had uninformative names (e.g., “hypothetical protein”, “unknown protein”). Using this approach, we estimate the total number of non-redundant, previously-characterized genes identified from this study is 14,695.

### ArrayStar Analyses

To determine which genes were exclusively expressed in one morphotype cDNA pool but not in the other, we re-mapped all 454 reads to the integrated transcriptome assembly or the *Danio rerio* genome (Fig. 5). This was accomplished using the QSeq software program module within ArrayStar (v.5; DNAStar, Madison, WI) employing either the integrated *Astyanax* transcriptome or the *Danio rerio* genome as the template. Both surface and cave form reads were then mapped to the template using the RPKM normalization method [64]. We performed an additional GO term analysis based on the 3,278 *Astyanax* genes that were identified from the *Danio* genome using ArrayStar software (Fig. 2C). We did not observe any genes, revealed through this analysis, that were not present in our integrated *Astyanax* transcriptome.

### Linkage Mapping

From our transcriptome analyses, we attempted to find informative SNP variation in 287 genes selected based on their genomic position in *Danio rerio* (see Fig. 3). Of 287 attempts, we found SNPs in 194 genes (∼68%). After eliminating 62 genes in which the SNPs were not suitable for sequencing, we attempted Sequenom iPlex sequencing to genotype 168 F_2_ individuals (derived by crossing surface fish × Pachón cavefish) for 132 markers (∼68%; Table S2). Genotypic analyses were carried out at the Broad Institute (Cambridge, MA; Table S3). Of the 132 markers we attempted to genotype, primer design failed for seven and no genotypic data was available. Genotypic data obtained for the remaining 125 markers were examined and we eliminated five markers that were not successfully genotyped in 25% or more of individuals and 20 markers that were uninformative (e.g., yielded an identical genotype in all individuals). The remaining 100 markers were loaded into JoinMap 3.0 (Kyazma) and linkage groups were created using a LOD score cut-off of 50.0. Of these 100 markers, 26 failed to group with at least one other marker and the remaining 74 formed 22 linkage groups (Table S4). Of the 22 linkage groups that were calculated, all but two (a total of nine markers) were successfully mapped, resulting in a final linkage map consisting of 65 markers spanning 20 linkage groups.

### Data Deposition

All Pachón cavefish and surface fish Roche/454 sequencing reads have been deposited to the National Center for Biotechnology Information (NCBI) Sequencing Read Archive (SRA Accession number: SRA062012; surface fish reads – SRX212200; Pachón cavefish reads – SRX212201).

## Supporting Information

Figure S1 **cDNA synthesis and normalization from RNA pools derived from surface dwelling and cave dwelling** ***Astyanax mexicanus*** **.** Total RNA was pooled from one male and one female adult Pachón cavefish, and one male and one female adult surface dwelling fish (*Astyanax mexicanus*). Total RNA samples were quality analyzed (A), and first-strand cDNA synthesis was carried out on poly(A)+ RNA isolated from the total RNA samples (B). cDNA was amplified by PCR using a proof-reading enzyme, and then normalized using one cycle of denaturation and reassociation (B). cDNA fragments in the range of 500–800 bp were extracted from an agarose gel and prepared for sequencing using the Roche GS FLX system (C).
(TIF)

Figure S2 **Integrated contigs enable rapid and simple identification of sequence polymorphisms between cave and surface morphotypes.** Each contig (horizontal blue line, A) in our transcriptome was assembled from multiple overlapping reads (black arrows, A) derived from both surface and cavefish cDNA. The gene *microspherule protein 1* (*mcrs1*) was assembled for a total length of 2,715 bp and demonstrated variable depths of sequence coverage across the assembled transcript (blue histogram, A). A SNP (red line, A) is evident from the consensus of multiple overlapping cave and surface sequences (yellow, A; B). Surface fish reads indicate the presence of a T nucleotide at position 1636, whereas the cavefish reads indicate a C at this position (red nucleotides, B). Using the SNP identification tool (C) in SeqMan (DNAStar, Methods), these polymorphisms can be easily identified and adapted for downstream genotypic analysis.
(TIF)

Figure S3 **An analysis of the relative position of orthologous** ***Astyanax*** **genes in the** ***Danio*** **genome.** We identified 7,806 contigs (∼34.5% of the total integrated assembly) in our *Astyanax* transcriptome that mapped to a known position in *Danio rerio*. The number of orthologous genes identified in our integrated transcriptome, organized by the chromosome number in which the homologous gene is found in *Danio rerio,* was uneven (A). The number of returned hits for a given chromosome is correlated to the number of genes that populate a given chromosome in *Danio* (r = 0.578, p = 0.002; B).
(TIF)

Figure S4 **Annotation of an integrated** ***Astyanax*** **transcriptome using Blast2GO.** Contigs that were most successfully annotated were ∼3,000–∼5,000 bp in length (A). The majority of sequences received an annotation score between ∼55–∼70 (B). The vast majority of sequences (>18,000) were coded as IEA (inferred from electronic annotation); (C). Remaining blast hits were distributed across the following evidence codes: ND (no biological data available), IMP (inferred from mutant phenotype), ISS (inferred from sequence or structural similarity), IDA (inferred from direct assay), TAS (traceable author statement), ISO (inferred from sequence orthology), IPI (inferred from physical interaction), IGI (inferred from genetic interaction), NAS (non-traceable author statement), EXP (inferred from experiment), IC (inferred by curator), IEP (inferred from expression pattern) and ISA (inferred from sequence alignment; C).
(TIF)

Table S1 **Catalog of BlastX identities of 22,596 contigs assembled from an integrated** ***Astyanax*** **transcriptome.**
(XLS)

Table S2 **Genotypic marker information used for linkage analysis in** ***Astyanax*** **.**
(XLS)

Table S3 **Gene marker sequences and primers used for genotypic analysis of a Pachón × surface F_2_ pedigree.**
(XLS)

Table S4 **Table of linkage data for markers derived from an integrated** ***Astyanax*** **transcriptome.**
(XLS)
