# Supplementary figures and images for: An Integrated Transcriptome-Wide Analysis of Cave and Surface Dwelling Astyanax mexicanus
Source: PLoS One. 2013 Feb 6;8(2):e55659. doi: 10.1371/journal.pone.0055659 (PMC3566029; doi:10.1371/journal.pone.0055659)

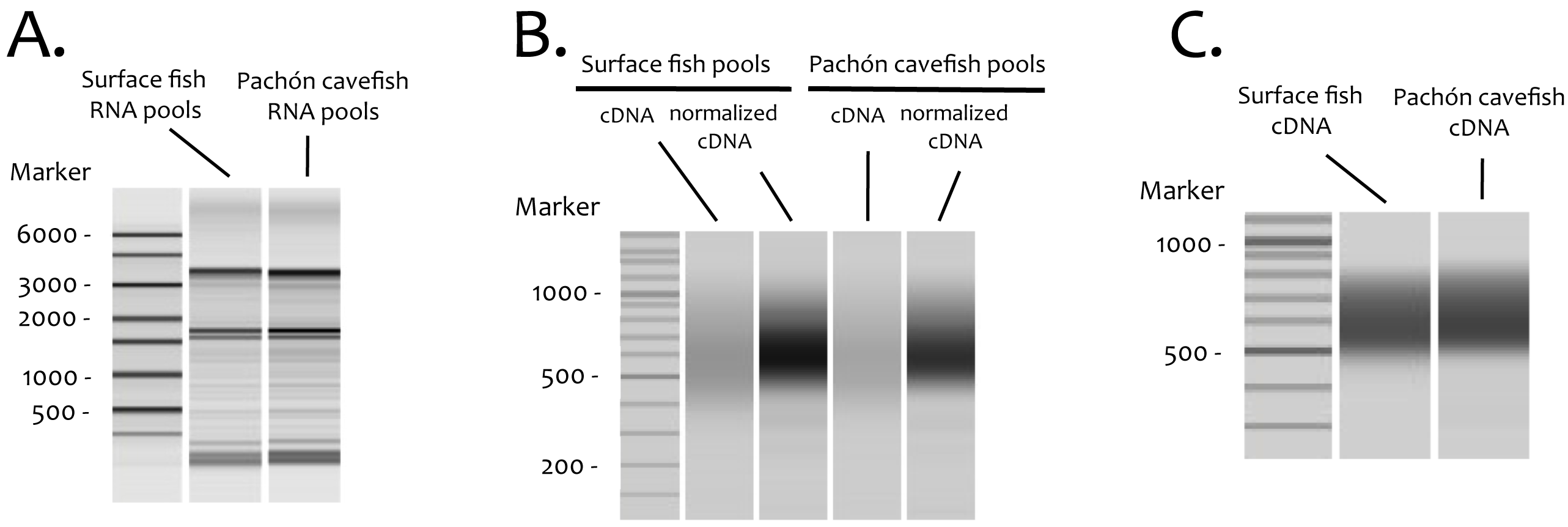

Supplement: Figure S1 — cDNA synthesis and normalization from RNA pools derived from surface dwelling and cave dwelling Astyanax mexicanus . Total RNA was pooled from one male and one female adult Pachón cavefish, and one male and one female adult surface dwelling fish (Astyanax mexicanus). Total RNA samples were quality analyzed (A), and first-strand cDNA synthesis was carried out on poly(A)+ RNA isolated from the total RNA samples (B). cDNA was amplified by PCR using a proof-reading enzyme, and then normalized using one cycle of denaturation and reassociation (B). cDNA fragments in the range of 500–800 bp were extracted from an agarose gel and prepared for sequencing using the Roche GS FLX system (C). (TIF) [file pone.0055659.s001.tif]

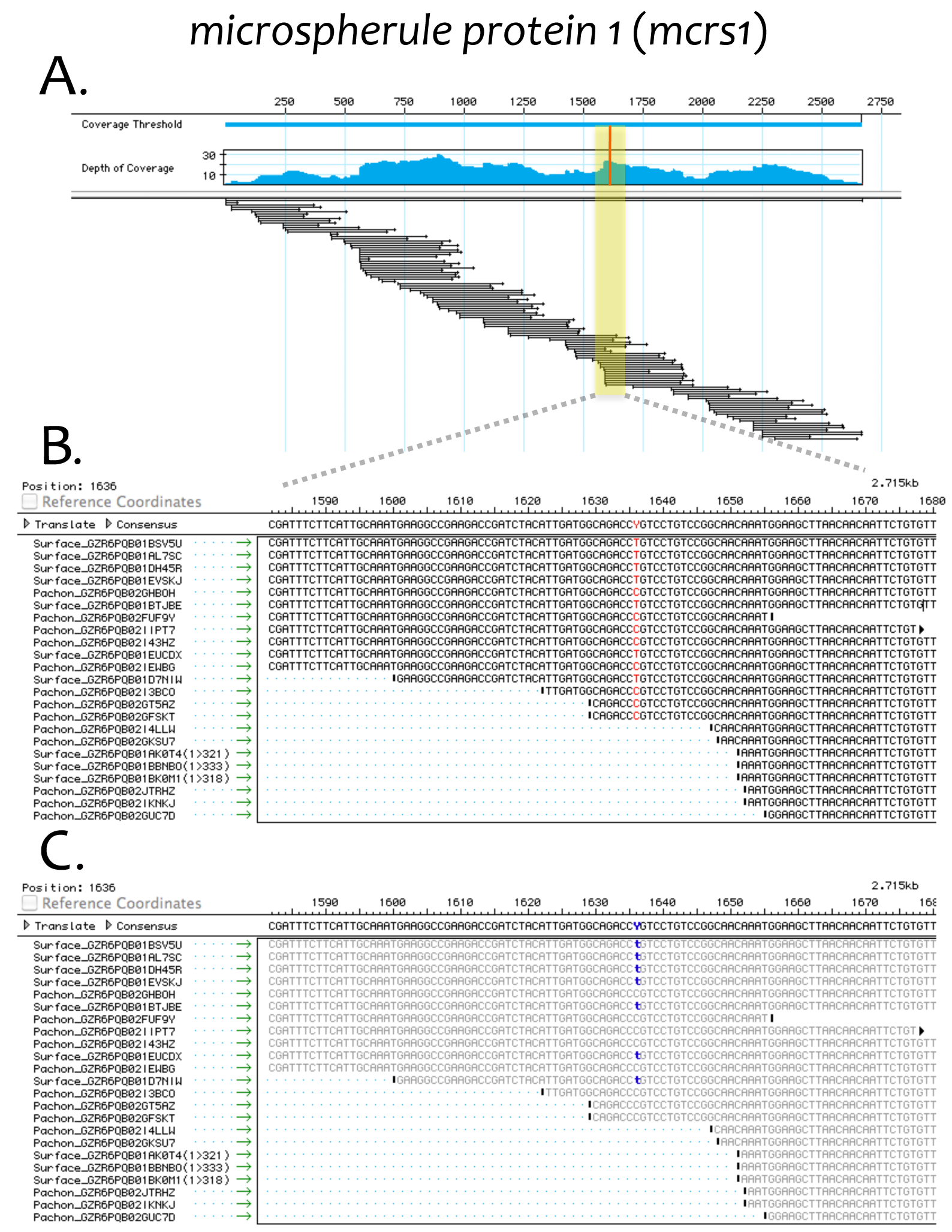

Supplement: Figure S2 — Integrated contigs enable rapid and simple identification of sequence polymorphisms between cave and surface morphotypes. Each contig (horizontal blue line, A) in our transcriptome was assembled from multiple overlapping reads (black arrows, A) derived from both surface and cavefish cDNA. The gene microspherule protein 1 (mcrs1) was assembled for a total length of 2,715 bp and demonstrated variable depths of sequence coverage across the assembled transcript (blue histogram, A). A SNP (red line, A) is evident from the consensus of multiple overlapping cave and surface sequences (yellow, A; B). Surface fish reads indicate the presence of a T nucleotide at position 1636, whereas the cavefish reads indicate a C at this position (red nucleotides, B). Using the SNP identification tool (C) in SeqMan (DNAStar, Methods), these polymorphisms can be easily identified and adapted for downstream genotypic analysis. (TIF) [file pone.0055659.s002.tif]

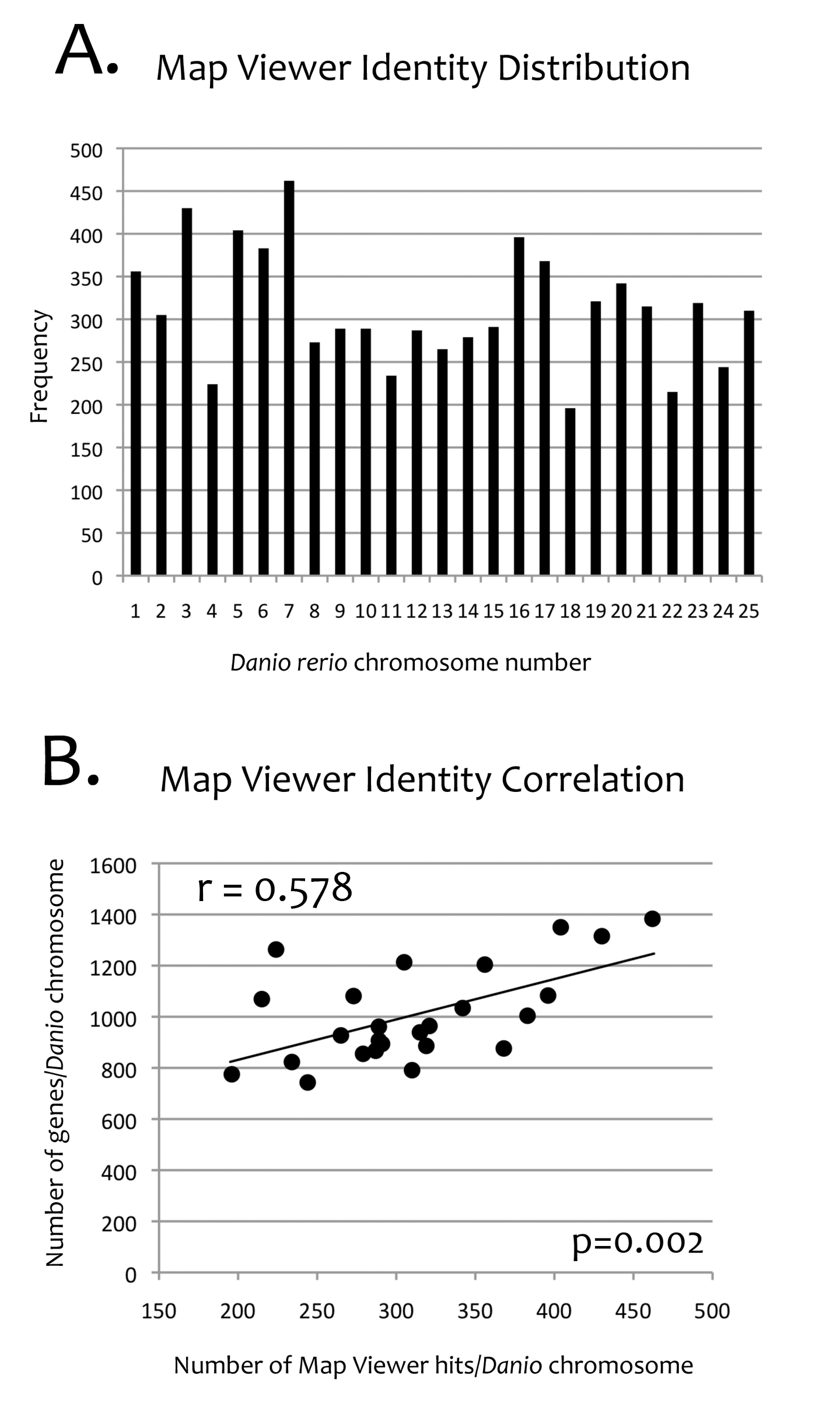

Supplement: Figure S3 — An analysis of the relative position of orthologous Astyanax genes in the Danio genome. We identified 7,806 contigs (∼34.5% of the total integrated assembly) in our Astyanax transcriptome that mapped to a known position in Danio rerio. The number of orthologous genes identified in our integrated transcriptome, organized by the chromosome number in which the homologous gene is found in Danio rerio, was uneven (A). The number of returned hits for a given chromosome is correlated to the number of genes that populate a given chromosome in Danio (r = 0.578, p = 0.002; B). (TIF) [file pone.0055659.s003.tif]

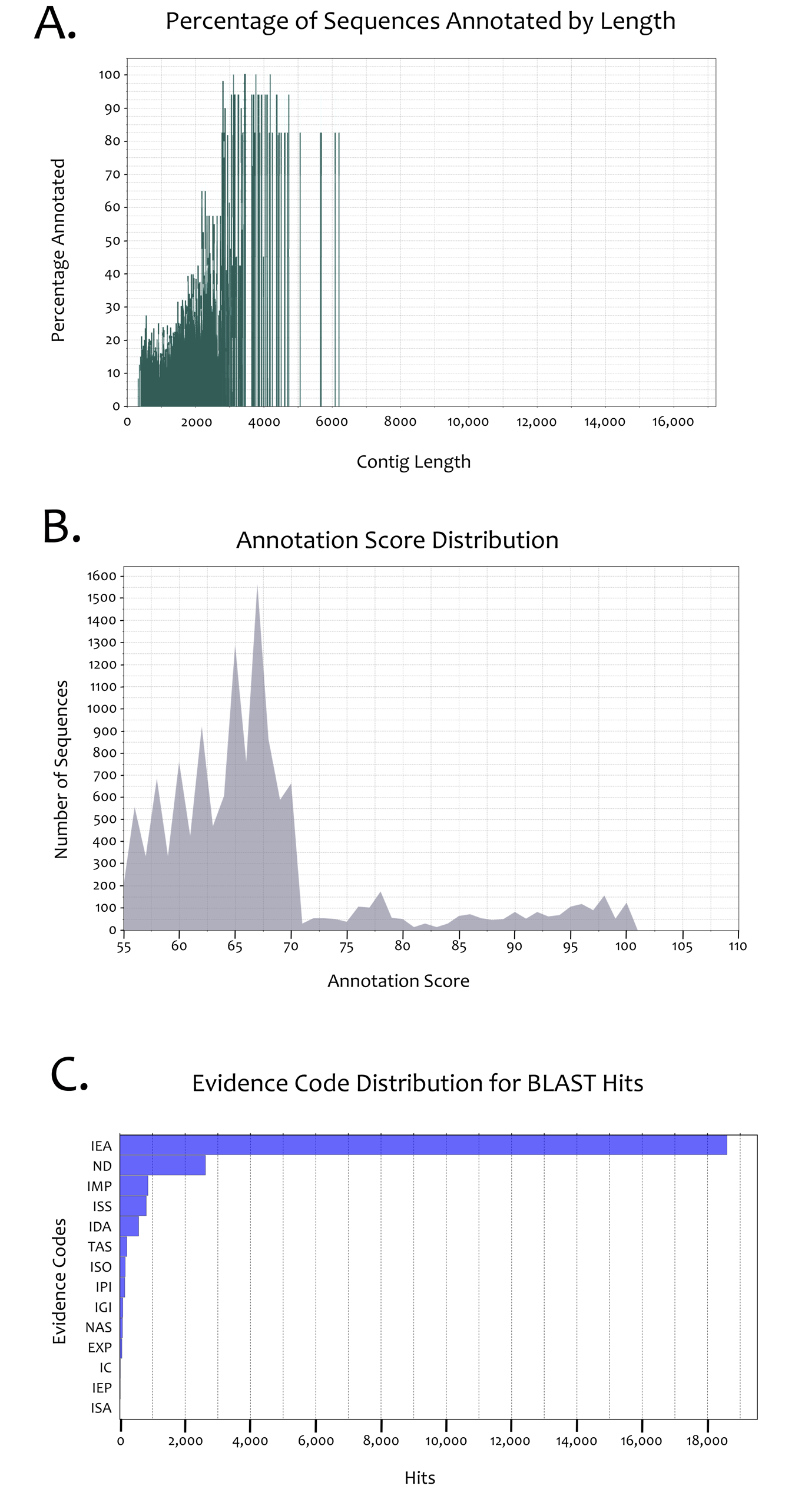

Supplement: Figure S4 — Annotation of an integrated Astyanax transcriptome using Blast2GO. Contigs that were most successfully annotated were ∼3,000–∼5,000 bp in length (A). The majority of sequences received an annotation score between ∼55–∼70 (B). The vast majority of sequences (>18,000) were coded as IEA (inferred from electronic annotation); (C). Remaining blast hits were distributed across the following evidence codes: ND (no biological data available), IMP (inferred from mutant phenotype), ISS (inferred from sequence or structural similarity), IDA (inferred from direct assay), TAS (traceable author statement), ISO (inferred from sequence orthology), IPI (inferred from physical interaction), IGI (inferred from genetic interaction), NAS (non-traceable author statement), EXP (inferred from experiment), IC (inferred by curator), IEP (inferred from expression pattern) and ISA (inferred from sequence alignment; C). (TIF) [file pone.0055659.s004.tif]
